# The Influence of Long-Acting Somatostatin Analogs on ^68^Ga-DOTATATE Uptake in Patients With Neuroendocrine Tumors

**DOI:** 10.1097/RLU.0000000000004776

**Published:** 2023-07-22

**Authors:** Youssef Chahid, Khaled Hashimi, Ewoudt M.W. van de Garde, Heinz-Josef Klümpen, N. Harry Hendrikse, Jan Booij, Hein J. Verberne

**Affiliations:** From the Departments of ∗ Radiology and Nuclear Medicine; † Clinical Pharmacy, Amsterdam UMC, University of Amsterdam, Amsterdam; ‡ Division of Pharmacoepidemiology and Clinical Pharmacology, Department of Pharmaceutical Sciences, Utrecht University, Utrecht; § Department of Medical Oncology, Amsterdam UMC, University of Amsterdam; ∥ Department of Radiology and Nuclear Medicine, Amsterdam UMC, Vrije Universiteit Amsterdam, Amsterdam, the Netherlands.

**Keywords:** ^68^Ga-DOTATATE, PET/CT, neuroendocrine tumors, somatostatin receptor imaging, somatostatin analogs

## Abstract

A high SUV_max_ tumor-to-liver ratio (TLR) of ^68^Ga-DOTATATE can be used to select patients with neuroendocrine tumors (NETs) for peptide receptor radionuclide therapy (PRRT). In addition, an SUV_max_ TLR ≥ 8.1 is associated with increased progression-free survival in NET patients treated with somatostatin analogs (SSAs). To avoid a theoretical interaction, several guidelines recommend performing PET/CT just before the monthly administration of long-acting SSAs. We aimed to investigate the effect of SSA on the SUV_max_ of ^68^Ga-DOTATATE in patients with NET and to identify independent predictors for high SUV_max_ TLR.

**Patients and Methods:**

For this retrospective study, 192 ^68^Ga-DOTATATE PET/CT scans of 165 patients without (n = 115) and with (n = 77) SSA (octreotide or lanreotide) in the 3 months before PET/CT were collected and reviewed. The effect of SSA on SUV_max_ values was analyzed by a maximum likelihood mixed model.

**Results:**

Patients with SSA had a significantly higher median SUV_max_ TLR than patients without SSA (4.7 [IQR], 3.1–7.7) versus 3.2 [IQR, 2.0–5.4]; *P* < 0.001). Multivariable logistic regression analysis showed that SSA use was an independent predictor for SUV_max_ TLR ≥ 8.1 (odds ratio, 2.91; 95% confidence interval, 1.26–6.72; *P* = 0.012).

**Conclusions:**

Our data suggest that higher SSA concentrations do not have a negative effect on ^68^Ga-DOTATATE uptake in tumor lesions. In addition, we found that only SSA use was associated with SUV_max_ TLR ≥ 8.1. Our results are consistent with previously conducted studies and in line with the recently published guideline that suggests that the relatively recent use of SSA does not necessitate any delay in ^68^Ga-DOTATATE PET/CT imaging.

Neuroendocrine tumors (NETs) are a heterogeneous group of rare tumors, with an increasing incidence of approximately 1.1 to 7.0 per 100,000 subjects in the United States in 1973 and 2012, respectively.^1^ Although NET can arise from any neuroendocrine cell throughout the body, the gastrointestinal tract and pancreas (GEP-NET) are the most affected organs.^1^ Approximately 70%–100% of all NETs are characterized by the overexpression of somatostatin receptor subtypes 2 (SSTR-2) and 5 (SSTR-5) on these tumor cells.^2^ Because of the high expression of SSTR, synthetic somatostatin analogs (SSAs) with high affinity for SSTR-2 and SSTR-5 are used for symptom control and to improve progression-free survival (PFS). Initially, SSAs were limited in their clinical use by their half-life of several minutes.^3^ The subsequently developed slow-release depot injections overcame that problem and significantly improved patient convenience. In Europe and the United States, 2 long-acting SSAs are approved for the treatment of GEP-NET: octreotide (Sandostatin LAR; Novartis, Switzerland) and lanreotide (Somatuline; Ipsen, France).^4,5^

The SSTR overexpression in NET makes these tumors suitable candidates for ^68^Ga-DOTATATE (^68^Ga-DOTA-octreotate) PET/CT imaging. Indeed, the radiotracer ^68^Ga-DOTATATE has a high affinity, especially for SSTR-2, and can be used for staging, restaging, identification of unknown primary tumor location, and selection of patients for peptide receptor radionuclide therapy (PRRT).^6^ PRRT with ^177^Lu-DOTATATE (7.4 GBq ^177^Lu-DOTATATE every 8 weeks [4 cycles] plus 30 mg octreotide every 4 weeks) has been shown to improve the PFS and quality of life compared with high-dose SSA (60 mg octreotide every 4 weeks) in patients with metastatic midgut NET.^7,8^ The SUV_max_ of ^68^Ga-DOTATATE can be used as a predictive marker to select patients for PRRT because the effectiveness of PRRT correlates to an SUV_max_ > 16.4 and an SUV_max_ tumor-to-liver ratio (TLR) > 2.2.^9^ Because an SUV_max_ TLR ≥ 8.1 is associated with a longer PFS in patients with GEP-NET, this parameter can also be used to predict the effectiveness of SSA.^10^

However, it is well known that SSA and ^68^Ga-DOTATATE bind preferentially to SSTR-2 in NET. To avoid a theoretical SSTR-2 drug interaction, the manufacturer and several guidelines recommend performing ^68^Ga-DOTATATE PET/CT just before the scheduled monthly administration of the SSA.^6,11–13^ In recent publications, including an updated guideline and a systematic review by Morland et al, it has been suggested that the interval between administrations of SSA may be less crucial for patients receiving stable doses of long-acting SSA than previously believed.^14,15^ This is based on data from some small studies, suggesting that this potential interaction of SSA has no effect on the diagnostic outcome of ^68^Ga-DOTATATE PET/CT.^16–20^ Unfortunately, these studies were too small to perform a multivariable analysis on patient and tumor characteristics to identify independent factors associated with high SUV_max_ TLR (ie, ≥8.1) in patients with NET.

Therefore, the primary aim of this study was to investigate the effect of long-acting SSA on the SUV_max_ of ^68^Ga-DOTATATE in tumor lesions in a larger patient population diagnosed with NET. In addition, potential independent predictors of a high SUV_max_ TLR were evaluated.

## PATIENTS AND METHODS

### Patient Population and Data Extraction

This study was retrospectively conducted at the Amsterdam University Medical Centers (Amsterdam UMC), University of Amsterdam, Amsterdam, the Netherlands. The design of the study was evaluated by the Medical Ethics Assessment Committee of the Amsterdam UMC and was approved (ie, due to the retrospective nature of the study, patient consent was waived; the official letter of Medical Ethics Assessment Committee is available on request). Patients with NET were eligible to be included if they had received ^68^Ga-DOTATATE PET/CT during the period from May 2016 until January 2023.

Patients were divided into 2 groups based on SSA use. The first group of patients was not treated with SSA. The second group consisted of patients who had at least 1 dose of long-acting SSA (octreotide or lanreotide) administered in the 3 months before ^68^Ga-DOTATATE PET/CT. This period of 3 months was chosen because of the pharmacokinetic profile of the octreotide depot, which has a long and slow drug release. The concentration of octreotide is stable during the first weeks after administration, but after approximately 6 weeks, the concentration of octreotide decreases slowly, concomitant with the terminal degradation phase of the polymer matrix of the depot.^4^ However, it is not known how long the depot of octreotide will remain active. For this reason, we chose a period of 3 months, but we also performed a subgroup analysis of recent SSA users (ie, patients who used SSA in the 3 weeks before ^68^Ga-DOTATATE PET/CT) compared with nonrecent SSA users (ie, patients who used SSA between 3 weeks and 3 months before PET/CT). To evaluate independent factors that may be associated with high SUV_max_ TLR, we collected the following data from the electronic health records database: sex, age, body mass index (BMI), primary tumor location, primary tumor resection, WHO NET grade, tumor stage, Ki-67 values, injected dose of ^68^Ga-DOTATATE, injected amount peptide of ^68^Ga-DOTATATE, treatment with ^177^Lu-DOTATATE, and treatment with SSA (type SSA, dose, and last administration before PET/CT imaging). To estimate the serum SSA concentration during ^68^Ga-DOTATATE PET/CT, we used the pharmacokinetics data from the respective Summaries of Product Characteristics (SmPCs).^4,5^

### Labeling of ^68^Ga-DOTATATE

Labeling of ^68^Ga-DOTATATE was performed using a kit preparation method. First, 1.1 mL ^68^Ga-solution in 0.1 M HCl was generated from a ^68^Ge/^68^Ga generator (GalliAd; IRE-Elit, Belgium). Then, 50 μg DOTATATE (ABX, Germany) was dissolved in 3.0 mL of sodium acetate buffer dilution, which was made from 5.0 mL European Pharmacopeia sodium acetate buffer solution pH 4.5 (Fischer Chemical, United Kingdom) and 95.0 mL NaCl 0.9% (B. Braun, Germany), and added to the 1.1 mL ^68^Ga solution. The solution was heated for 7 minutes at 95°C. Quality control was performed using a radio high-performance liquid chromatography system. The radiochemical purity of the final product was at least 95%.

### PET/CT Procedure

PET/CT image acquisition was performed 45 to 60 minutes after IV administration of approximately 40 μCi/kg ^68^Ga-DOTATATE. Patients were instructed to drink at least 500 mL of water and to urinate before the scan. Until October 2017, the Gemini Time-of-Flight PET/CT scanner (Philips, the Netherlands) was used. Diagnostic contrast-enhanced (100 mL IV Ultravist 300; Bayer, Germany) CT was performed in the portal phase from the skull base to the thigh (120 kV, 150 mAs, 16 × 1.5 collimation, 0.8013 pitch). Then, PET acquisition was performed with a scan time of 2.5 minutes per bed. From October 2017, a Biograph mCT Flow PET/CT scanner (Siemens, Germany) equipped with an enhanced axial field of view (TrueV) scanner was used. Diagnostic contrast-enhanced CT was performed in the portal phase with automatic modulation in current and voltage. Reference values were set at 120 kV and 160 mA, 128 × 0.6 collimation, and 0.9 pitch. CT was performed after administration of 90 mL IV iodinated contrast medium (Xenetix 350; Guerbet, France). PET was performed with continuous bed motion at 1.5 mm/s.

For both scanners, CT data were used for PET attenuation correction and PET data reconstruction. Iodinated contrast medium was only administered on clinical indication and when there were no contraindications for administration.

### Image Analysis

The ^68^Ga-DOTATATE PET/CT images were analyzed using Hybrid Viewer software (version 5.1.0; Hermes Medical Solutions, Sweden). The SUV_max_ was calculated with a spherical volume of interest (VOI) tool. The VOI was drawn manually in the primary tumor, the liver (ie, physiological uptake), and the left psoas major muscle (ie, background). The VOI for the primary tumor was dependent on the tumor size. A fixed VOI of 4 cm^3^ was used for the right lower lobe of the liver, and a fixed VOI of 2 cm^3^ was used for the middle of the left psoas major muscle. If multiple tumor lesions were present, for practical reasons, the lesion with the highest ^68^Ga-DOTATATE uptake was chosen to calculate the SUV_max_. Independent of the scanner used, we determined the following ^68^Ga-DOTATATE PET/CT parameters:

▪ SUV_max_ tumor-to-background ratio (SUV_max_ TBR) = SUV_max_ tumor/SUV_max_ psoas major muscle
▪ SUV_max_ liver-to-background ratio (SUV_max_ LBR) = SUV_max_ liver/SUV_max_ psoas major muscle
▪ SUV_max_ tumor-to-liver ratio (SUV_max_ TLR) = (SUV_max_ tumor − SUV_max_ psoas major muscle)/(SUV_max_ liver − SUV_max_ psoas major muscle)

Although SUV_max_ TLR is most clinically relevant due to the physiological high uptake and the frequent presence of metastases in the liver, an SUV_max_ TLR ≥ 8.1 appears to be associated with a longer PFS in patients treated with SSA and was therefore also included in our multivariable analysis.^10,19^

### Statistical Analysis

Patient, tumor, and medication characteristics were evaluated using descriptive statistics. The results are shown as frequencies with percentages for categorical variables and means ± standard deviations (SDs) or medians with interquartile ranges (IQRs) for continuous variables. Pearson χ^2^ exact test or Fisher exact test was used for categorical variables, and the unpaired *t* test or Mann-Whitney *U* test was used for quantitative variables. The original SUV_max_ values follow a log-normal distribution. The effect of SSA on the log-transformed SUV_max_ values was analyzed by fitting a mixed model as implemented in IBM SPSS Statistics (version 28; IBM, USA). This mixed model uses a compound symmetry covariance matrix and is fitted using maximum likelihood. In the absence of missing values, this method results in the same *P* values as multiple comparisons tests (eg, repeated-measures analysis of variance) that are less able to deal with missing values. Variables with a *P* value below 0.20 in the univariate analysis were included in the multivariable logistic regression models. All statistical tests were 2-tailed, and a *P* value below 0.05 was considered statistically significant. Odds ratios (ORs) of significant predictors of SUV_max_ TLR ≥ 8.1 are presented with 95% confidence intervals (CIs).

## RESULTS

### Study Population

In total, 165 patients were included in this study: 138 patients with 1 PET/CT scan (n = 88 patients without SSA, n = 50 patients with SSA) and 27 patients with 2 PET/CT scans (1 scan without SSA and 1 scan with SSA). The patients’ characteristics during the PET/CT scans (n = 115 with SSA and n = 77 without SSA) are summarized in Table 1. The majority of the patients were male (n = 108, 56.3%), and 63 patients were treated with SSA. Approximately 90% of primary tumor locations were the small bowel (n = 80, 41.7%), pancreas (n = 78, 40.6%), rectum (n = 9, 4.7%), and stomach (n = 5, 2.6%). A total of 55 patients received lanreotide, and 22 patients received octreotide in the 3 months before ^68^Ga-DOTATATE PET/CT. The median last SSA injection was 30 (IQR, 19–35) days before PET/CT imaging. Patients with SSA were more likely to have been diagnosed with metastases and had more follow-up scans than patients without SSA therapy. Patients with and without SSA use did not differ in WHO NET grade, tumor stage, the amount of administered ^68^Ga-DOTATATE activity (μCi/kg), or ^68^Ga-DOTATATE peptide amount (ng/kg).

**TABLE 1:** Patient Characteristics of the Study Population

Characteristic	115 PET/CT Scans Without SSA (%)	77 PET/CT Scans With SSAs (%)	*P*
Sex			0.553∥
Male	67 (58.3)	41 (53.2)	
Female	48 (41.7)	36 (46.8)	
Age, y†	63.2 ± 11.7	65.1 ± 10.2	0.260#
BMI, kg/m^2^†	25.8 ± 4.9	25.3 ± 5.2	0.538#
Ki-67 value (%)*	3.0 (1.0–7.5)	3.3 (1.0–7.5)	0.953¶
Primary tumor location			0.117**
Small bowel	40 (34.8)	40 (51.9)	
Pancreas	55 (47.8)	23 (29.9)	
Rectum	5 (4.3)	4 (5.2)	
Stomach	3 (2.6)	2 (2.6)	
Other/unknown	12 (10.4)	8 (10.4)	
Primary tumor resection			0.135∥
Resected	41 (35.7)	36 (46.8)	
Not resected‡	74 (64.3)	41 (53.2)	
WHO NETs grade			0.349**
I	52 (45.2)	33 (42.9)	
II	53 (46.1)	39 (50.9)	
III	6 (5.2)	1 (1.3)	
Unknown	4 (3.5)	4 (5.2)	
Tumor stage			0.185**
I	9 (7.8)	4 (5.2)	
II	24 (20.9)	16 (20.8)	
III	20 (17.4)	5 (6.5)	
IV	5 (4.3)	6 (7.8)	
Unknown	57 (49.6)	46 (59.7)	
Metastasis			<0.001**
Yes	87 (75.7)	75 (97.4)	
No	28 (24.3)	2 (2.6)	
PET/CT			<0.001**
Initial	56 (48.7)	0 (0.0)	
Follow-up	59 (51.3)	77 (100.0)	
PET/CT scanner			0.568∥
Philips	22 (19.1)	12 (15.6)	
Siemens	93 (80.9)	65 (84.4)	
Activity, μCi/kg†	56 ± 12	58 ± 12	0.214#
Peptide, ng/kg†	147 (105–218)	124 (101–175)	0.063¶
^177^Lu-DOTATATE therapy			0.069**
Yes	4 (3.5)	8 (10.4)	
No	111 (96.5)	69 (89.6)	
SSAs treatment			
Lanreotide	NA	55 (71.4)	
Octreotide	NA	22 (28.6)	
Last injection, days*,§	NA	30 (19–35)	
Estimated concentration, ng/mL*	NA	2.03 (1.42–3.89)	

*Median (IQR).

†Mean ± standard deviation.

‡Including patients with an unidentified primary tumor.

§Days between the last administration of SSAs and ^68^Ga-DOTATATE PET/CT.

∥Pearson χ^2^ exact test.

¶Mann-Whitney *U* test.

#Unpaired *t* test.

**Fisher exact test.

NA, not applicable.

### ^68^Ga-DOTATATE Uptake

The data in Table 2 show that patients with SSA in the 3 months before ^68^Ga-DOTATATE PET/CT had a significantly higher median SUV_max_ TBR than patients without SSA (32.8 [IQR, 23.2–49.3] vs 26.3 [IQR, 16.9–44.1]; *P* < 0.001). The median SUV_max_ LBR was not significantly different between the groups (*P* = 0.435). The median SUV_max_ TLR was significantly higher for patients with SSA than for patients without SSA (4.7 [IQR, 3.1–7.7] vs 3.2 [IQR, 2.0–5.4]; *P* < 0.001).

**TABLE 2:** SUV_max_ Values of PET/CT Scans With SSA Compared With Those Without SSA

Characteristic	115 PET/CT Scans Without SSA (%)*	77 PET/CT Scans With SSAs (%)*	*P*†
SUV_max_ TBR	26.3 (16.9–44.1)	32.8 (23.2–49.3)	<0.001
SUV_max_ LBR	9.1 (6.9–10.9)	7.0 (5.6–8.3)	0.435
SUV_max_ TLR	3.2 (2.0–5.4)	4.7 (3.1–7.7)	<0.001

*Median (IQR).

†Linear mixed model.

TBR, tumor-to-background ratio; LBR, liver-to-background ratio; TLR, tumor-to-liver ratio.

### Recent Use of SSA

Patients treated with recent SSA (ie, in the 3 weeks before ^68^Ga-DOTATATE PET/CT) did not show significant differences in mean SUV_max_ TBR, SUV_max_ LBR, and mean SUV_max_ TLR compared with nonrecent SSA users. Patients without SSA treatment had a significantly lower mean SUV_max_ TLR than recent SSA users (3.2 [IQR, 2.0–5.4] vs 4.7 [IQR, 2.9–7.6]; *P* = 0.004) and nonrecent SSA users (3.2 [IQR, 2.0–5.4] vs 4.4 [IQR, 3.2–8.1]; *P* < 0.001). The mean SUV_max_ TLR was not significantly different between the recent and nonrecent SSA users (*P* = 0.932).

### Predictive Factors for SUV_max_ TLR ≥ 8.1

There were 29 patients with an SUV_max_ TLR ≥ 8.1 (Table 3). Univariate analysis showed that primary tumor location, tumor resection, and SSA treatment were associated with SUV_max_ TLR ≥ 8.1 (*P* < 0.20). Subsequent multivariate logistic regression analysis showed that only the use of SSA (OR, 2.91; 95% CI, 1.26–6.72; *P* = 0.012) was an independent predictor for SUV_max_ TLR ≥ 8.1.

**TABLE 3:** Univariate and Multivariable Logistic Regression Analysis of PET/CT Scans With SUV_max_ TLR ≥ 8.1

Characteristic	n	PET/CT Scans With SUV_max_ TLR ≥ 8.1 (%)	Univariate Analysis	Multivariate Analysis	
			*P*	Adjusted OR (95% CI)	*P*
Sex			0.899		
Male	108	16 (14.8)			
Female	84	13 (15.5)			
Age, y†	192	65.1 ± 10.8	0.537		
BMI, kg/m^2^†	192	25.7 ± 6.0	0.953		
Ki-67 value (%)*	192	3.5 (1.0–5.8)	0.730		
Primary tumor location			0.105∥		0.100
Pancreas	78	18 (23.1)			
Small bowel	80	8 (10.0)			
Rectum	9	1 (11.1)			
Stomach	5	1 (20.0)			
Other/unknown	20	1 (5.0)			
Primary tumor resection			0.140		0.099
Resected	77	8 (10.4)			
Not resected‡	115	21 (18.3)			
WHO NETs grade			0.587∥		
I	85	11 (12.9)			
II	92	16 (17.4)			
III	7	1 (14.3)			
Unknown	8	1 (12.5)			
Tumor stage			0.376∥		
I	13	0 (0.0)			
II	40	4 (10.0)			
III	25	1 (4.0)			
IV	11	2 (18.2)			
Unknown	103	22 (21.4)			
Metastasis			1.000∥		
Yes	162	25 (15.4)			
No	30	4 (13.3)			
PET/CT			0.839		
Initial	56	8 (14.3)			
Follow-up	136	21 (15.4)			
PET/CT scanner			0.328		
Philips	34	7 (20.6)			
Siemens	158	22 (13.9)			
Activity, μCi/kg†	192	56 ± 14	0.859		
Peptide, ng/kg†	192	151 (113–250)	0.277		
^177^Lu-DOTATATE therapy			0.697∥		
Yes	12	1 (8.3)			
No	180	28 (15.6)			
SSAs treatment			0.030		0.012
None	115	12 (10.4)		1	
SSAs	77	17 (22.1)		2.91 (1.26–6.72)	
SSAs concentration*§	77	3.74 (1.67–3.89)	0.253		

*Median (IQR).

†Mean ± standard deviation.

‡Including patients with an unidentified primary tumor.

§Estimate concentration (ng/mL) based on pharmacokinetics data from SmPCs.

∥Fisher exact test.

## DISCUSSION

We found that patients treated with SSA in the 3 months before ^68^Ga-DOTATATE PET/CT had a significantly higher median SUV_max_ TBR and significantly higher SUV_max_ TLR than patients without SSA. In addition, we showed that patients with recent use of SSA had no significant differences in median SUV_max_ TBR, median SUV_max_ LBR, or median SUV_max_ TLR compared with patients with nonrecent use of SSA.

We found a significantly higher median ^68^Ga-DOTATATE tumoral SUV_max_ in patients with SSA than in patients without SSA (32.8 vs 26.3; *P* < 0.001). A higher ^68^Ga-DOTATATE tumoral SUV_max_ was previously reported by Cherk et al^20^: in 21 patients after initiation of SSA therapy, 61% of 49 metastatic lesions had an increased SUV_max_ after SSA therapy compared with baseline values at 6 months (range, 2–12 months) before the start of SSA treatment. In addition, Aalbersberg and colleagues^19^ described a significant increase in the mean SUV_max_ in 190 tumor lesions in 31 patients 1 day after administration of lanreotide (21.64 ± 12.63 vs 20.96 ± 12.37; *P* = 0.034). Our results are in line with the findings of both studies and might be explained by the upregulation of SSTR-2 in NET. Froidevaux and coworkers^21^ indeed observed, after 7 days of continuous IV administration of SSA, 150% upregulation of SSTR-2 in SSTR-2 tumor cells in AR4-2J tumor-bearing SCID mice compared with controls. Nevertheless, this observation of SSTR-2 upregulation is still disputable because some studies found no significant differences in ^68^Ga-DOTATATE tumoral uptake after SSA treatment.^16–18^ However, one could wonder whether this difference in uptake of ^68^Ga-DOTATATE could also be explained by the functional state of the tumor, that is, expression of differences in hormonal hypersecretion, implying the clinical need for SSA. Although tempting as an explanation, this was not eminent from our retrospective data.

The median SUV_max_ TLR was also significantly higher in the SSA group. Although Ayati et al^16^ did not show significant differences in the mean SUV_max_ TLR, this observation is in line with the majority of the available literature.^17–20^ Kim and coworkers showed that an SUV_max_ TLR ≥ 8.1 is associated with a longer PFS in patients with GEP-NET treated with SSA.^10^ To our knowledge, our multivariable logistic regression analysis is the first to show that only the use of SSA (OR, 2.91; 95% CI. 1.26–6.72; *P* = 0.012) is independently associated with SUV_max_ TLR ≥ 8.1. An interesting approach to deliver higher doses of ^177^Lu-DOTATATE into tumors may be pretreatment with SSA. However, further research is needed to provide more insights and to test this postulate.

The steady-state SSA concentration will be achieved after 4 administrations of SSA when given every 4 weeks. The steady-state concentration of 30 mg octreotide is 2.6 ng/mL and remains relatively constant during the first 6 weeks.^4^ The steady-state concentration of 120 mg lanreotide is approximately 7.0 ng/mL and has a half-life of 23–30 days.^5^ The European guidelines recommend performing ^68^Ga-DOTATATE PET/CT just before the scheduled monthly administration of the SSA,^6,12,13^ that is, to wait until approximately 50% of the steady-state concentration of lanreotide.^5^ However, our data suggest that higher SSA concentrations do not have a negative effect on ^68^Ga-DOTATATE uptake in tumor lesions, which is in line with the suggestion of recently published guideline recommendations.^14^ We showed that patients with recent use of SSA had no significant differences in median SUV_max_ in tumors compared with patients with nonrecent use of SSA before PET/CT imaging. In line with our results, Galne and colleagues^17^ also showed no association of SUV_max_ with the interval of SSA administration before PET/CT. In fact, Aalbersberg et al^19^ showed only a slightly increased mean SUV_max_ in tumor lesions 1 day after compared with 1 day before administration of lanreotide (21.64 vs 20.96; *P* = 0.034). Based on the above published studies, our present findings, and the pharmacokinetic profile of SSA, we postulate that there is no need to delay ^68^Ga-DOTATATE PET/CT until just before the scheduled monthly administration of SSA. Continuation of long-acting SSA has several benefits, including no need to switch to short-acting SSA, clustering and performing ^68^Ga-DOTATATE PET/CT examinations at any time without regard to individual patient SSA administration schedules.

There are some limitations to this study, including its retrospective design. Second, different PET/CT scanners were used during the study period, making comparison of SUV_max_ somewhat cumbersome. However, variation between different PET/CT scanners reflects daily practice and is often an excepted limitation of multicenter clinical trials. To compensate for this limitation, we used the tumor-to-background and TLRs, reducing variation between different cameras and potentially allowing for extrapolation of our results to other different clinical settings.^22^ In addition, the presented SSA concentrations during the ^68^Ga-DOTATATE PET/CT are not measured but estimated based on the pharmacokinetics data from the respective SmPCs.

## CONCLUSIONS

This study shows that patients with long-acting SSA in the 3 months before ^68^Ga-DOTATATE PET/CT had a significantly higher median SUV_max_ TLR than patients without SSA. In addition, we did not find any differences in ^68^Ga-DOTATATE uptake between patients who recently used SSA and patients with SSA between 3 weeks and 3 months before PET/CT. Based on these data, in combination with the pharmacokinetic profile of SSA and the results of previously conducted studies, we do agree with the recently published suggestion that performing ^68^Ga-DOTATATE PET/CT just before the scheduled monthly administration of SSA is not a prerequisite for adequate patient management.

## ACKNOWLEDGMENT

The authors would like to thank Edwin Poel for his statistical support.
